# Environmental Heterogeneity and Phenotypic Divergence: Can Heritable Epigenetic Variation Aid Speciation?

**DOI:** 10.1155/2012/698421

**Published:** 2012-03-04

**Authors:** Ruth Flatscher, Božo Frajman, Peter Schönswetter, Ovidiu Paun

**Affiliations:** ^1^Institute of Botany, University of Innsbruck, Sternwartestraße 15, 6020 Innsbruck, Austria; ^2^Department of Systematic and Evolutionary Botany, University of Vienna, Rennweg 14, 1030 Vienna, Austria

## Abstract

The dualism of genetic predisposition and environmental influences, their interactions, and respective roles in shaping the phenotype have been a hot topic in biological sciences for more than two centuries. Heritable epigenetic variation mediates between relatively slowly accumulating mutations in the DNA sequence and ephemeral adaptive responses to stress, thereby providing mechanisms for achieving stable, but potentially rapidly evolving phenotypic diversity as a response to environmental stimuli. This suggests that heritable epigenetic signals can play an important role in evolutionary processes, but so far this hypothesis has not been rigorously tested. A promising new area of research focuses on the interaction between the different molecular levels that produce phenotypic variation in wild, closely-related taxa that lack genome-wide genetic differentiation. By pinpointing specific adaptive traits and investigating the mechanisms responsible for phenotypic differentiation, such study systems could allow profound insights into the role of epigenetics in the evolution and stabilization of phenotypic discontinuities, and could add to our understanding of adaptive strategies to diverse environmental conditions and their dynamics.

## 1. Introduction

Patterns and causes of biological variation have fascinated and challenged natural scientists for a long time. The Darwinian evolutionary theory highlights the importance of natural variation as raw material upon which selection processes can act, thereby increasing the fitness of locally adapted phenotypes [1]. Conceptual and technical developments since the late 19th century have greatly enhanced our understanding of some of the main mechanisms producing and maintaining biological variation, namely, genetic mutation and recombination [2]. However, natural selection acts upon phenotypic variation represented by the individual [3], which is delimited by its genetic constitution, but also shaped by its specific environment [4] and developmental processes [5]. The process of evolution is thus a result of complex interactions between various intrinsic and extrinsic factors [6].

Therefore, current evolutionary investigations should consider several levels of biological variation [7]. First, differences in the DNA sequence account for a great amount of biological variation: the genetic system defines the range of functional possibilities of each individual. However, these heritable differences translate into the phenotype only indirectly via the resulting RNA and protein products which mould the structure and function of an organism. Much progress has been made in recent years in identifying gene functions and candidate genes coding for important metabolic enzymes, but analyses of whole genomes remain a complex challenge. Even in organisms whose whole genome is sequenced, a large number of genes still remain uncharacterized [8]. The second important source of biological variation is fluctuation in rates of gene expression, resulting in phenotypic plasticity [9, 10]. Genes can be up- or downregulated in response to environmental conditions, such as temperature regimes or water supply, or intrinsic factors such as specific phenological or developmental stages [11]. This leads to temporary modifications of the phenotype, which are generally not passed on to the next generation [12, 13]. The third level, heritable epigenetic variation, via both specialized enzymology inducing structural modifications of the DNA (through DNA methylation, histone acetylation [14, 15]) and small interfering (si) RNA populations [16, 17], results in (meta) stable chromatin landscape differences. Epigenetic differences determine if and where particular genes or groups of genes are to be expressed, while the underlying DNA sequence remains identical [18]. Most of these differences are reversible developmental effects and they are part of the molecular processes underlying phenotypic plasticity in response to variation in the environment [19]. However, environmental change, severe stress or genomic shock events like hybridization or genome duplication can change the epigenetic configuration of an organism resulting in new phenotypes [20–26], and some of these alterations can be passed on to the next generations [27–30].

The molecular mechanisms underlying these components of phenotypic variation differ in their stability and in the time frames in which they confer phenotypic novelty. The genetic sequence is the most stable, evolving slowly through mutation and gradually accumulating changes over a large number of generations. In contrast, gene expression levels can be rapidly and continuously regulated within a very short time [11], much shorter than the generation length of an organism, and allow an almost instantaneous response of the individual to its environment within limits defined by its genetic constitution. Heritable epigenetic alterations act within an intermediate time horizon, since they can occur as an immediate and multilocus reaction to different kinds of external or intrinsic stimuli [23] but are not as ephemeral as plastic gene regulation and can affect the following generations [18].

It has long been established that mutations in DNA sequence are the primary raw material for evolutionary change [2]. The involvement of environmental influences in generating heritable biological variation is still debated [13, 22], as is the necessity of extending our modern evolutionary synthesis [31]. Accumulating evidence indicates that modifications of epigenetic signals are correlated with phenotypic variation within and among species [25, 32–34], placing epigenetic differentiation even in a macroevolutionary context. Latest developments regarding the potential role of phenotypic plasticity in driving diversification and speciation have been discussed elsewhere (e.g., [13, 35]). We are hereafter focusing on the impact of heritable epigenetic variation on the process of evolution and propose a research plan to address its evolutionary significance.

## 2. Potential Impact of Heritable Epigenetic Variation on Evolution

Empirical studies have demonstrated high levels of epigenetic variation within natural populations [25, 36–41]. While experiments have shown that environmental conditions can override epigenetic signals (e.g., [26, 42, 43]) and increase this variation, few recent studies indicate that natural selection can act directly or indirectly on epigenetic variation [25, 38, 39, 44], potentially leading to evolutionary divergence and adaptation. Altogether, epigenetic information provides an additional source of natural variation, which may be particularly important for survival of small populations lacking genetic variability [45] and/or occupying a fragmented landscape. Selectable epigenetic variation can enable genetically depauperate lineages to adapt [46] until genetic assimilation occurs (i.e., when environmentally induced phenotypic variation becomes fixed by secondary genetic control, e.g., after deamination of methylated cytosine to thymine [13, 47]). Thus, heritable epigenetic variation could pave the way for genetic adaptation.

The epigenetic sources of variation can be stochastic epimutations, but a major part of the epigenetic variation is triggered by stress or changes in the environment [3, 22, 48], that is, under circumstances when new phenotypes could be crucial for survival. Moreover, if conditions return to their original state, spontaneous back-mutation of epialleles can restore original phenotypes (e.g., in position-effect variegation [27]). In the light of epigenetic variation, the involvement of the environment in evolution becomes twofold: as a stimulant of variation and as the selector of adaptive variation.

At the interface between genotype and environment, the overall rate of epimutations is often much higher than that of genetic mutations [49], resulting in a more dynamic level of variation. Novel epigenetic modifications may originate simultaneously in several individuals in a population under stress, which will facilitate fixation. Despite the potentially high loss of epigenetic novelties by epigenetic reset [19], epimutations can reach equilibrium frequencies within populations rapidly, over less than a dozen generations if the environmental stress is maintained long enough [28]. In stark contrast to the expected incidence of genetic mutations, environmental fluctuations can trigger multiple epimutations in the same individual. This renders fast ecological adaptation affecting (complex) adaptive traits more plausible [50]. Hence, recombination is not necessarily a prerequisite for adaptive change, if the latter is driven from the epigenetic level. In addition, epigenetic mechanisms may partly defy well-understood population processes, such as allelic drift (due to potential maintenance of relatively constant epiallelic frequencies through environmental influence). Being more flexible and dynamic than DNA sequence information, variation in epigenetic signals could therefore act as major driving force in rapid adaptive processes.

Epigenetic variation can have extensive consequences, even in the absence of genetic variability [45, 50, 51]. Epigenetics may introduce, or reinforce in a back-coupling process with environmental stimuli, major changes that lead to strong phenotypic differentiation [52] until becoming a real reproductive barrier. Most phenotypic differences between species are genetically controlled, but epigenetic inheritance can be of particular importance for the initial development of phenotypic divergence [25]. If adaptive and maintained long enough, phenotypic discontinuities can become genetically locked and trigger species divergence [53]. Modelling studies suggest that epigenetic variation can promote population divergence by facilitating adaptive peak shifts, reducing genetic barriers represented by fitness valleys in the adaptive landscape [47]. Therefore, epigenetic novelties have been one of the mechanisms put forward for saltational speciation [29, 54], but empirical data is not yet available to support or reject such a hypothesis.

## 3. A Research Idea

Recently developed tools, in combination with traditional methods, can shed light on the complex interactions between genotype, epigenotype, and environment, and test for their individual contribution to phenotypic divergence and evolution. Evolutionary biologists could address the evolutionary relevance of heritable epigenetic polymorphisms by targeting closely related ecotypes or species (hereafter types) that show phenotypic differentiation without apparent genome-wide genetic divergence. Such types could be identified, for example, within asexual lineages or descendants of recent adaptive radiation events. We suggest a multifaceted research plan using an array of molecular techniques and field experiments to investigate whether epigenetics is involved in speciation by triggering phenotypic diversification.

### 3.1. Phenotypic Differentiation

As speciation is facilitated by the process of divergence, the first question to be addressed should be whether phenotypic variation in the study group is discrete or continuous. Phenotypic variation is a common feature of populations and species, and only a discontinuity in this variation may indicate incipient divergence and the onset of isolating mechanisms. Therefore, various morphological, anatomical, and physiological traits among populations of different types should be compared to test whether the types form well defined, distinct groups or whether the extreme phenotypes are linked by individuals with intermediate traits or combinations of characters. In addition, measurements and observations of environmental characteristics (e.g., microclimate, geology, soil, biological interactions) could identify limiting environmental factors, and relate them to anatomical, morphological, and physiological specializations.

**Figure 1:**
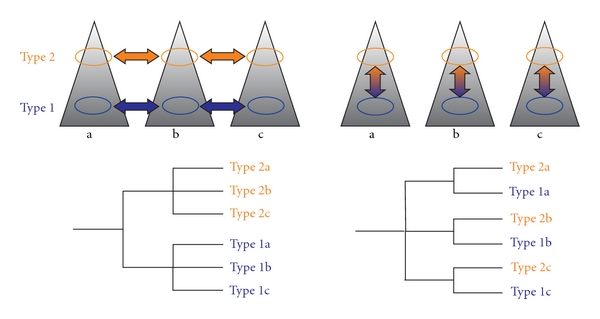
Putative relationships between populations of closely related alternative types (here exemplified with altitudinal differentiation), which lack apparent genome-wide divergence. Below the reflection of the relationships in hypothetical phylogenies is given. Left, single origin of each type, followed by dispersal to other geographical areas. Right, recurrent evolution of the types in several geographic regions under environmental influence.

If main discontinuities in phenotypic variation separate populations along type boundaries (e.g., by morphology or habitat preference), the uniformity within each group and constant difference between the groups might suggest a single origin of each type and subsequent dispersal (Figure 1). However, this seems rather unlikely in absence of genome-wide genetic divergence among the types. An alternative scenario could invoke repeated migration and iterative in situ formation of each type in alternative environments, with very strong and almost identical selection pressures acting upon different populations of each of the types.

### 3.2. Genetic and Epigenetic Differentiation

Singular versus multiple origin of each type should be tested by investigating the extent and structure of genome-wide genetic and epigenetic divergence within and among populations of both types. If populations cluster genetically in disagreement to the type (possibly determined by other factors, e.g., by geographic proximity), it may be hypothesized that their differentiation is underlaid by epigenetic mechanisms and that types have evolved several times in parallel. Alternatively, local high rates of gene flow combined with strong selection at a few adaptive genetic loci could hypothetically produce a similar pattern of highly porous genomes [55]. In such a case, a small number of adaptive (outlier) genetic loci of large effect should be responsible for the observed phenotypic differentiation. Outlier analyses [56–58] of genetic profiles provided, for example, by DNA fingerprinting techniques such as RAD (restriction site associated DNA) sequencing [59], microsatellites, or AFLP (amplified fragment length polymorphism [60]), could help identifying these loci or closely linked genomic regions. Positive selection will shape at target loci a significantly higher differentiation between populations of the alternative types than the genome-wide bulk of loci, while loci under purifying selection will show much lower differentiation [61]. On the other hand, if individuals of each group share type-specific epigenetic patterns and/or mRNA transcripts, differentiation could be mediated either by overall differences in the epigenome or by a few epialleles. 

As epigenetic variation is not detectable in genomic surveys of sequence variation, dedicated investigations have to be employed to address it. In recent years, a variety of genome-wide approaches, including techniques involving next-generation sequencing, have been developed to comparatively profile epigenetic patterns in nonmodel organisms [62, 63]. Cost-effective comprehensive methods include, for example, fractioning the DNA using C_0_t filtration [64, 65] to enrich low-copy regions (mostly genes and their promoters) and sequence this genomic subsample by employing next generation methods and bisulfite sequencing. The latter is a process that converts unmethylated cytosines to uracils, which will then appear as thymines after sequencing [66]. Third-generation DNA sequencers, like the recently released single molecule real-time (SMRT) DNA sequencer could be employed for direct detection of DNA methylation [67] and thus enable much more profound study of both model and nonmodel epigenomes. Alternatively, genome-wide DNA methylation could be studied using isoschizomers [68, 69]. Similarly as for genetic dataset(s), the epigenetic information could be searched for general patterns of differentiation and for signatures of selection on individual (epi)loci [25, 44]. This should clarify if ecological and/or morphological divergence is dependent on just a few loci controlling traits for local adaptation, or if it is triggered by extensive differences. As the alternative types thrive in different environments, the selective pressures and their magnitude may vary across populations. Epigenetic signals will most often suffer from imperfect heritability; therefore, stronger selection will be needed to produce patterns that will be detected as outliers by statistical approaches.

To infer broad, genome-wide regulatory variation, in-depth quantitative gene expression analyses using next-generation sequencing (RNA-seq, [70–72]) could be performed searching for loci with significant expression differences between individuals of different types after growing them under uniform conditions to reduce the momentary-dependent noise in rates of expression. In addition, targeting posttranscriptional regulation, small RNA profiles could be compared using an smRNA-seq approach [63, 73, 74]. The different data types can finally be integrated in functional analyses (i.e., gene annotations) to identify correlated components that are part of the same regulatory network.

### 3.3. Heritability of Phenotypic Plasticity and Habitat Specificity

If the molecular basis of phenotypic differentiation and/or adaptation to divergent environments is identified within epigenetic rather than DNA sequence divergence, the next research step would be to investigate how stable the phenotypic divergence is. This will also help to assess the stage of speciation in which the group is at present. While facilitating population divergence and speciation [35], nonheritable phenotypic plasticity will trigger speciation only if the environmental conditions are stably different in the alternative localities [35] and gene flow is either infrequent or strongly opposed by natural selection. On the other hand, in the case of heritable phenotypic divergence that is fully stable even in the alternative environment, epigenetically triggered adaptation may have been already assimilated in the genetic code.

Reciprocal transplant experiments together with attempts to grow the different types under the same environment across several generations (i.e., between three and five as a minimum requirement) should be installed to determine the extent of phenotypic plasticity, and the ability of the different types to cope with altered environmental conditions. Growing individuals of the alternative types in a uniform environment across several generations may reveal the heritability of morphological and ecological characteristics within each of the types (“nature versus nurture”) [75]. Comparatively investigating relevant (epi)loci in transplanted individuals versus controls will pinpoint those patterns that are immediately disrupted by the environment, and those that persist or, alternatively, are not under the influence of the relevant limiting environmental differences. Integrating this information and comparing morphological, anatomical and physiological traits supplemented by a set of fitness components among transplants and controls will define the links between genotype, epigenotype and phenotype, together with providing additional information on the patterns of selection and their targets.

According to the mechanisms underlying the observed differentiation, at least two possible outcomes can be anticipated. If the morphological and/or ecophysiological differences are triggered by continuous but nonheritable responses to local environments (i.e., as a reaction norm [76]), there should be no phenotypic differences between the progeny of the two types when reared and grown under the same conditions. Such a scenario will not (yet) be relevant for speciation. On the other hand, if heritable epigenetic differences are involved, phenotypic divergence between individuals of the types should at least partly be retained in a common environment. In the latter case the morphology, anatomy, and physiological properties of the transplanted individuals should reflect their origin rather than their current environment. This may go to the extreme that individuals are maladapted and do not survive under alien environmental conditions.

The result of these experiments could simultaneously allow for inferring evolutionary and population dynamics within the study group. If individuals of alternative types can adapt phenotypically to the habitat of the other and develop the habitat-specific syndromes following transplant experiments, the possibility of frequent gene flow between populations of both types should be considered. This might as well explain the lack of overall differentiation, as it prevents lineage sorting and hampers or slows down speciation. On the contrary, low fitness (i.e., poor performance and high mortality) of individuals in the native habitat of the alternative type may point to a differentiation that is strong enough to prevent gene flow between populations. In this case, we may be observing a process of ongoing speciation, where differentiation starts at the epigenetic level, triggering profound changes leading to segregation in terms of habitat, phenology, and/or biological interactions. Divergent selection may reinforce this environmentally induced specialization/niche segregation and bring about reproductive isolation. This will eventually result in virtual isolation of gene pools, and ultimately give way to stronger overall differentiation by accumulation of genetic differences due to the stochastic effects of drift.

## 4. *Heliosperma pusillum* Group: An Example of an Appropriate Study System

**Figure 2:**
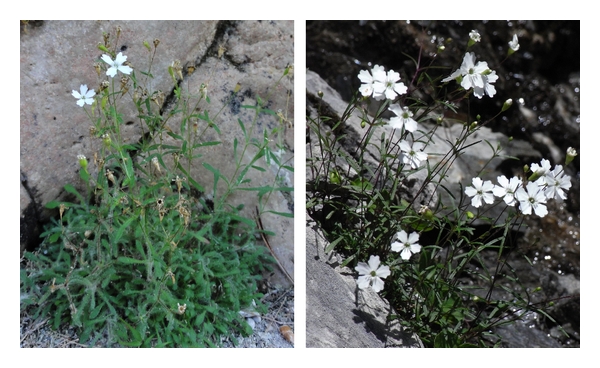
Low-elevation *Heliosperma veselskyi* and high-elevation *H. pusillum* are differentiated morphologically and ecologically. Particularly conspicuous is the dense indumentum of sticky glandular hairs on *H. veselskyi* in comparison to the glabrous leaves and stems of *H. pusillum* (Photographs: M. Sonnleitner).

**Figure 3:**
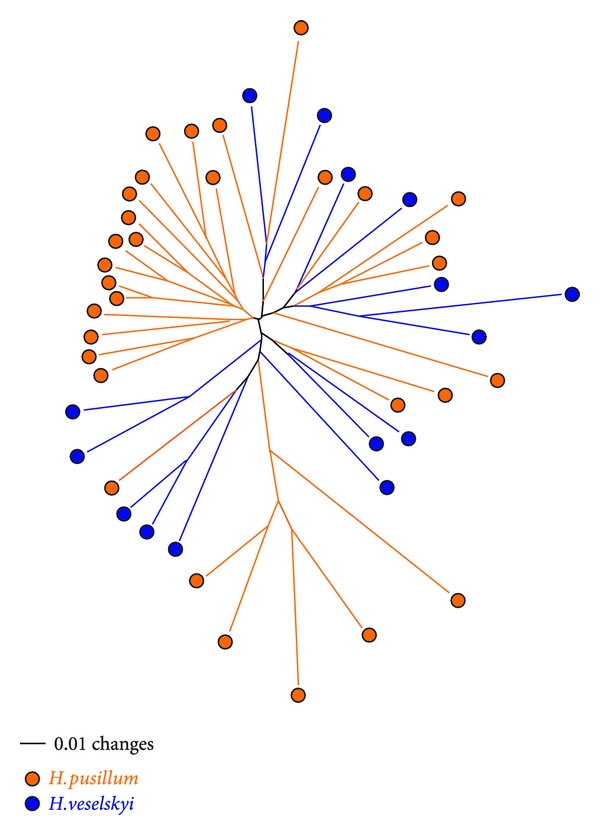
Genetic analyses do not support separation of higher-altitude *Heliosperma pusillum* (orange) and lower-altitude *H. veselskyi* (dark blue), but rather indicate an inextricable relationship between the two taxa. Unrooted neighbor joining tree based on Nei-Li distances calculated with PAUP from AFLP profiles [77].

*Heliosperma pusillum* and allied taxa from the carnation family (Caryophyllaceae) contain a variety of morphologically different taxa (Figure 2) with distinct ecology, which are altitudinally or geographically isolated, but genetically intermixed (Figure 3) and do not represent independent evolutionary lineages [78]. Molecular phylogenetic studies based on AFLPs [77] and sequences of several nuclear and chloroplast regions [77–79], show that genetic divergence within the group is generally shallow, many taxa seem to be polyphyletic, and geographically allied taxa often share the same genetic constitution. We hypothesize that they either (i) represent fixed ecotypes, that is, differ subtly in their DNA coding regions with major phenotypic effects, or (ii) result from middle- to short-term adaptive (epigenetic) processes, perhaps under the influence of the environment and independent of actual changes in DNA sequence. All of them are perennial caespitose herbs that inhabit rocky habitats and shallow caves in mountain ranges of southern Europe [78, 80], mostly on calcareous substrates. 

Different authors [78, 81] have subdivided this complex into two ecologically and morphologically distinct groups of taxa: a higher elevation group occurring in damp, open habitats and among rocks above the timberline and a lower elevation group inhabiting canyons and gorges as well as shallow caves and cliff overhangs with rather dry soils, high atmospheric moisture and poor light conditions below the timberline. The higher elevation group, including *H. albanicum*, *H. pudibundum, *and *H. pusillum* s.str., differs from the lower elevation group by narrower, glabrous or sparsely hairy leaves and often unicellular glands as well as longer seed papillae [78, 81]. By contrast, plants of lower elevations share a denser indumentum with long multicellular glandular hairs and are often sticky (Figure 2). Generally, morphological variation is much higher in the lower elevation group, which contains several narrowly distributed taxa [78, 82]. Most of them are endemics of the Balkan Peninsula; only *H. veselskyi* is restricted to the southeastern Alps. The origin and evolution of the lower and higher elevation groups and the relationships between them are still poorly understood. Recent molecular phylogenetic studies [78] (see also Figure 3) indicate that neither higher nor lower elevation groups are actually monophyletic, but rather inextricably intermingled with each other, indicating that one of the groups evolved multiple times from the other. Mechanisms involved in the phenotypic diversification of the two groups, the morphological convergence within each group, and the stability of this phenotypic divergence remain unknown, but preliminary evidence suggests that morphological features remain constant in a common garden, at least in the first generation. The *H. pusillum *complex is suitable for (epi) genomic and transcriptomic analyses, because all taxa have a relatively small genome (1C = 1.32 pg [83]) and so far no polyploid cytotypes have been found (2*n* = 2*x* = 24). In addition, they can be easily grown from seeds and have short generation times, which make them optimally suitable for common garden and transplantation studies.

## 5. Synthesis and Outlook

Although the possibility of epigenetic inheritance has now been established [7, 18, 27, 30, 84] and we are increasingly understanding the full extent of its role in producing phenotypic variation [19, 25, 39, 40], little research has been done to systematically study the role of heritable epigenetic variation for speciation. Incorporation of epigenetics into evolutionary models and empirical studies is only now starting to be attempted (e.g., [28, 49]); however, more empirical information from natural populations is needed for accurate modelling of epigenetic dynamics. Indeed, the prevalence of alternative stable epialleles in natural populations, and their significance to phenotypic divergence, ecological interactions and selection in real-world contexts remain too little explored [3, 41, 53]. The limited relevant data available indicate a stochastic nature of epigenetic variation, which is continuously being shaped by the influence of the environment, and further tuned through natural selection [25, 38, 39]. Therefore, the epigenetic aspect of natural variation may contribute to evolution in a fashion similar to genetics, but much more rapidly. Implying heritability of adaptive (i.e., selected) traits, epigenetic inheritance is not a contradiction of the Darwinian evolutionary synthesis [31], but rather a complex augmentation of the classic view on genetic inheritance, particularly as genotype and epigenotype interact to produce a broad array of short- and long-term heritable combinations.

The recently available possibility to profile the epigenome and transcriptome of nonmodel organisms in a high-throughput manner [62, 63, 85] enables thorough investigation of some of the most challenging hypotheses in a modern evolutionary framework, such as achieving and maintaining stable divergence through epigenetic differences. The acquired knowledge also impacts several related domains, from conservation to theoretical evolutionary biology. Investigating recent adaptive radiations with epigenetic markers may be particularly informative. Most traits of ecological significance tend to be continuous or quantitative and appear to be governed by many genes, each of little effect, but with cumulative power [86], resulting in a complex picture of factors and mechanisms acting upon the phenotype. Using appropriate study systems it is now possible to interrogate the links between ecological divergence and many regulatory alterations of small effect or singular major epigenetic switches. In addition, such investigations are expected to pinpoint new loci that are sensitive to epigenetic modification and unravel information on the rates of spontaneous epimutations in natural populations and their stability over time.

Currently accumulating data will offer valuable clues on the establishment of broad regulatory determinants of functional diversity in natural populations. The early evidence we currently hold urges complementing our gene- and genome-centred evolutionary view with a substantial consideration of epigenetic factors when seeking to understand population processes that drive adaptation and divergence [3, 53, 87]. Using modern technologies, future research will identify the exact molecular mechanisms triggering relevant phenotypic divergence and reproductive isolation. We will soon be able to infer the corresponding selection pressures that are responsible for the presence of a particular individual/a particular species in its specific habitat. Understanding how new plant species form and adapt to novel ecological niches is crucial to advance our knowledge of evolutionary processes active at the population level driving adaptation and speciation. An increased knowledge of organismic adaptation strategies is also of outstanding importance in the current context of widespread environmental challenges. It may be a key for predicting effects of climate change and managing biodiversity in a sustainable manner.
